# Hyperglycemia-induced metabolic compensation inhibits metformin sensitivity in ovarian cancer

**DOI:** 10.18632/oncotarget.4556

**Published:** 2015-06-19

**Authors:** Lacey M. Litchfield, Abir Mukherjee, Mark A. Eckert, Alyssa Johnson, Kathryn A. Mills, Shawn Pan, Viji Shridhar, Ernst Lengyel, Iris L. Romero

**Affiliations:** ^1^ Department of Obstetrics and Gynecology, Gordon Center for Integrative Science, University of Chicago, Chicago, Illinois, USA; ^2^ Department of Laboratory Medicine and Experimental Pathology, Mayo Clinic Cancer Center, Rochester, Minnesota, USA

**Keywords:** ovarian cancer, metformin, hyperglycemia, glycolysis, c-Myc

## Abstract

Increasing interest in repurposing the diabetic medication metformin for cancer treatment has raised important questions about the translation of promising preclinical findings to therapeutic efficacy, especially in non-diabetic patients. A significant limitation of the findings to date is the use of supraphysiologic metformin doses and hyperglycemic conditions *in vitro*. Our goals were to determine the impact of hyperglycemia on metformin response and to address the applicability of metformin as a cancer therapeutic in non-diabetic patients. In normoglycemic conditions, lower concentrations of metformin were required to inhibit cell viability, while metformin treatment in hyperglycemic conditions resulted in increased glucose uptake and glycolytic flux, contributing to cell survival. Mechanistically, maintenance of c-Myc expression under conditions of hyperglycemia or via gene amplification facilitated metabolic escape from the effects of metformin. *In vivo*, treatment of an ovarian cancer mouse model with metformin resulted in greater tumor weight reduction in normoglycemic vs. hyperglycemic mice, with increased c-Myc expression observed in metformin-treated hyperglycemic mice. These findings indicate that hyperglycemia inhibits the anti-cancer effects of metformin *in vitro* and *in vivo*. Furthermore, our results suggest that metformin may elicit stronger responses in normoglycemic vs. hyperglycemic patients, highlighting the need for prospective clinical testing in patients without diabetes.

## INTRODUCTION

Drug repurposing is a paradigm in which a Food and Drug Administration-approved drug is utilized for a new indication, accelerating the timeline and decreasing the cost of drug development [1]. Such a strategy is particularly attractive for the treatment of ovarian cancer, where platinum-based therapies have been in use since the late 1970s and improvements in survival have been minimal [2]. In an effort to improve survival, a VEGF inhibitor, bevacizumab, has recently been added to the conventional carboplatin/paclitaxel regimen. This combination results in modest improvements in progression-free survival [3, 4], but comes at a cost of approximately $58,050 per patient [5]. There is a clear need for the identification of economical, safe, and effective drugs for the treatment of ovarian cancer, potentially through the repurposing of drugs used for alternative indications.

In recent years, epidemiological studies have identified an association between use of a common diabetes treatment, metformin (1,1-dimethylbiguanide hydrochloride), and improved survival in diabetics with gynecologic cancers (reviewed in [6]), including ovarian [7, 8]. Metformin use has also been associated with improved survival in hepatocellular [9], colorectal [10, 11], prostate [12], and pancreatic [13] cancers. These studies, however, provide little information on the potential benefits of metformin for cancer patients without diabetes. Metformin functions in the treatment of type 2 diabetes by increasing insulin sensitivity and reducing serum glucose concentrations, largely through inhibition of hepatic gluconeogenesis and enhanced muscular glucose uptake [14]. If the anti-cancer benefits of metformin in diabetic patients occur as a result of a systemic reduction in hyperglycemia and hyperinsulinemia, survival benefits may not translate to non-diabetic patients [15, 16].

In addition to its systemic effects, metformin directly alters the energy balance in cells through inhibition of complex I of the mitochondrial electron transport chain, resulting in an increase in the AMP/ATP ratio. Subsequent activation of AMP-activated protein kinase (AMPK) works to restore energy balance in the cell by inhibiting energy-consuming processes, including fatty acid and protein synthesis [14, 15]. As recently reviewed [6], preclinical studies demonstrate that metformin inhibits tumor growth and alters metabolism in gynecologic cancers. In ovarian cancer, metformin treatment has been shown to activate (phosphorylate) AMPK, inhibit fatty acid and protein synthesis, and result in cell death [17, 18]. However, these studies, and most *in vitro* studies in other cancer types, used doses of metformin (10-40 mM) which would not be achievable in patients [15, 16]. Whether these increased concentrations of metformin are truly necessary to achieve anti-cancer effects, or if they are merely a result of the inherent artificiality and hyperglycemic nature of *in vitro* experiments, has been a source of recent study [19-22].

In the present study, we sought to determine the impact of lower doses of metformin on ovarian cancer under normo- and hyperglycemic conditions. To approach this, we used multiple ovarian cancer cell lines, primary cells from a patient with ovarian cancer, and a syngeneic mouse model to test the hypothesis that hyperglycemic conditions inhibit the anti-cancer effects of metformin by allowing for a compensatory increase in glycolysis and escape from the energetic stress induced by metformin treatment. Overall, our goals were to determine if anti-ovarian cancer effects can be attained with lower doses of metformin and to begin to address the clinical question of the applicability of metformin as a cancer therapeutic for patients without diabetes.

## RESULTS

### The cytotoxicity of metformin is impaired in hyperglycemic conditions

**Figure 1:**
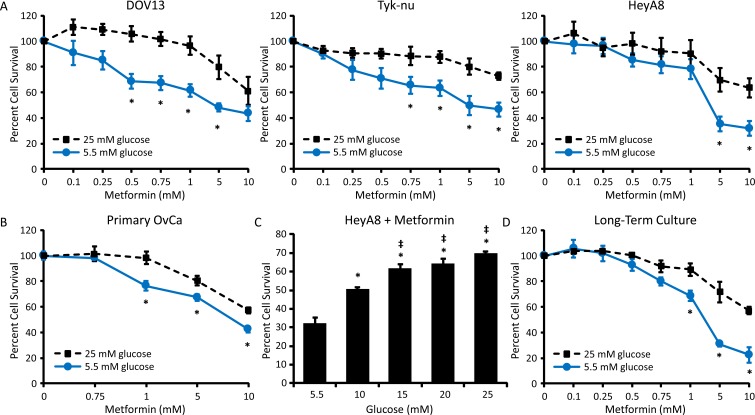
Hyperglycemia decreases the cytotoxic effect of metformin **A.** DOV-13, Tyk-nu, and HeyA8 cells were treated with 0-10 mM metformin in normoglycemic (5.5 mM glucose) or hyperglycemic (25 mM glucose) conditions for 72 h and viability was measured using an MTT assay. Values are mean ± SEM, *n* ≥ 3. *p < 0.05 *vs*. same metformin concentration in 25 mM glucose. **B.** MTT viability assay of primary ovarian cancer cells treated with 0-10 mM metformin in normoglycemic (5.5 mM glucose) or hyperglycemic (25 mM glucose) conditions for 72 h. Values are mean ± SEM, *n* = 5 in one experiment. *p < 0.05 *vs*. same metformin concentration in 25 mM glucose. **C.** MTT assay of HeyA8 cells treated with 5 mM metformin for 72 h in the presence of increasing glucose concentrations (5.5-25 mM glucose). Values are mean ± SEM, *n* ≥ 3. *p < 0.05 *vs*. treatment in 5.5 mM glucose, ‡*p* < 0.05 *vs*. treatment in 10 mM glucose. **D.** MTT assay of HeyA8 cells cultured for 2 weeks in normoglycemic or hyperglycemic conditions and treated with 0-10 mM metformin for 72 h. Values are mean ± SEM, *n* = 3. *p < 0.05 *vs*. same metformin concentration in 25 mM glucose.

To determine the effect of glycemic conditions on the response to metformin, three ovarian cancer cell lines were treated with a range of metformin concentrations and cell viability was evaluated. In normoglycemic conditions (5.5 mM glucose [23]), metformin treatment resulted in a dose-dependent inhibition of DOV13, Tyk-nu, and HeyA8 cell viability. In contrast, in “standard” cell culture conditions, which are hyperglycemic (25 mM glucose [23]), metformin's cytotoxic effect was suppressed (Figure 1A). A similar response was observed using primary human ovarian cancer cells isolated from ascites, with increased cytotoxicity noted following metformin treatment in normoglycemic conditions (Figure 1B). Additionally, a dose-response relationship was noted, with increasing concentrations of glucose resulting in decreasing metformin cytotoxicity in HeyA8 cells (Figure 1C). To ensure that increased metformin response in normoglycemic conditions was not mediated solely by the acute reduction in glucose levels in the cell culture media, long-term cultures were performed. Here, HeyA8 cells were cultured in media containing 5.5 or 25 mM glucose for two weeks. The media was changed daily and glucose levels were monitored to ensure that stable glucose concentrations were maintained. Following long-term exposure to normo- or hyperglycemic conditions, cells cultured in 5.5 mM glucose continued to demonstrate an enhanced response to metformin, as determined by effects on cell viability (Figure 1D).

### Higher doses of metformin are necessary to activate AMPK in hyperglycemic conditions

**Figure 2:**
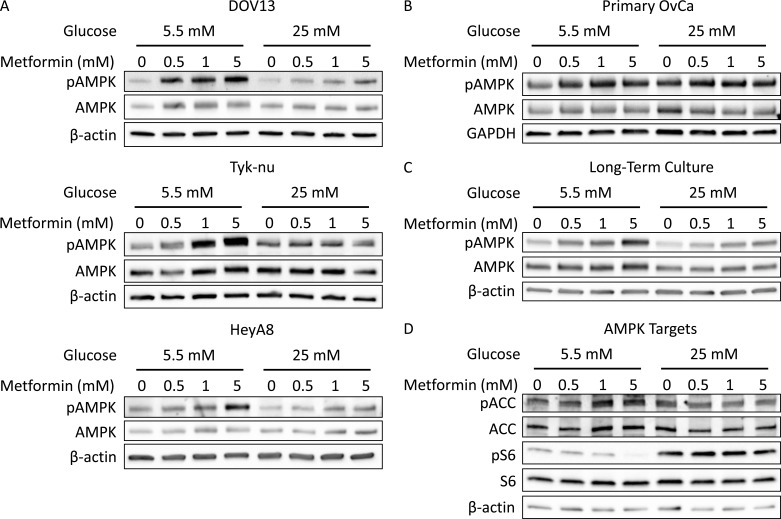
Hyperglycemia inhibits activation of AMPK by metformin **A.** Western blots of pAMPK Thr172 (62 kDa), AMPK (62 kDa), and β-actin (42 kDa) in DOV13, Tyk-nu, and HeyA8 cells treated with 0-5 mM metformin in normoglycemic (5.5 mM glucose) or hyperglycemic (25 mM glucose) conditions for 24 h. **B.** Western blot of pAMPK Thr172 (62 kDa), AMPK (62 kDa), and GAPDH (37 kDa) in primary ovarian cancer cells treated with 0-5 mM metformin in normoglycemic or hyperglycemic conditions for 24 h. **C.** Western blot of pAMPK Thr172 (62 kDa), AMPK (62 kDa), and β-actin (42 kDa) in HeyA8 cells cultured for 2 weeks in normoglycemic or hyperglycemic conditions and treated with 0-5 mM metformin for 24 h. **D.** Western blot of pACC Ser79 (280 kDa), ACC (265 kDa), pS6 Ser240/244 (32 kDa), S6 (32 kDa), and β-actin (42 kDa) in HeyA8 cells treated with 0-5 mM metformin in normoglycemic or hyperglycemic conditions for 24 h.

One hypothesized mechanism by which metformin inhibits cancer growth is through phosphorylation and activation of AMPK [14, 15]. As a measure of metformin response, three ovarian cancer cell lines and primary ovarian cancer cells were treated with a range of metformin concentrations in normo- or hyperglycemic conditions and the phosphorylation of AMPK (pAMPK) at Thr172 was analyzed. Metformin treatment at doses ≤ 5 mM led to a dose-dependent increase in pAMPK in all three cell lines and primary cells in media containing 5.5 mM glucose, while there was only minimal AMPK activation (phosphorylation) in media containing 25 mM glucose (Figure 2A–2B). A similar effect was noted when cells underwent long-term exposure to differential glucose conditions. Increased activation of AMPK by metformin was noted in cells cultured for two weeks in 5.5 mM glucose as compared to 25 mM glucose (Figure 2C).

To test whether glycemic conditions altered the effects of metformin on targets downstream of AMPK activation, a key mediator of fatty acid synthesis (acetyl-CoA carboxylase (ACC)) and a marker of protein synthesis (ribosomal protein S6) were evaluated [24]. Phosphorylation (inactivation) of ACC at Ser79 was increased by metformin treatment in 5.5 mM glucose, but not in 25 mM glucose (Figure 2D). Likewise, phosphorylation of S6 at Ser240/244 was suppressed by metformin treatment in 5.5 mM glucose, but not in 25 mM glucose (Figure 2D). These data suggest that in normoglycemic conditions low doses of metformin are able to activate AMPK, resulting in the inhibition of anabolic processes, including fatty acid and protein synthesis.

### Response to phenformin is also suppressed by hyperglycemia

**Figure 3:**
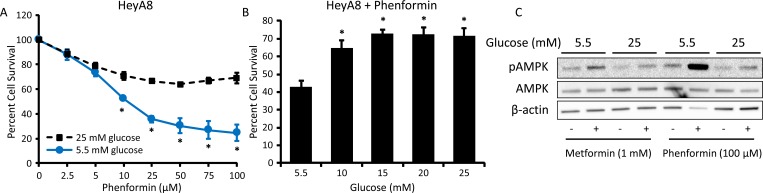
Hyperglycemia inhibits the effects of phenformin on cell viability and AMPK activation **A.** MTT assay of HeyA8 cells treated with 0-100 μM phenformin in normoglycemic (5.5 mM glucose) or hyperglycemic (25 mM glucose) conditions for 72 h. Values are mean ± SEM, *n* = 3. *p < 0.01 *vs*. same phenformin concentration in 25 mM glucose. **B.** MTT assay of HeyA8 cells treated with 25 μM phenformin for 72 h in the presence of increasing glucose concentrations (5.5-25 mM glucose). Values are mean ± SEM, *n* = 5. *p < 0.01 *vs*. treatment in 5.5 mM glucose. **C.** Western blot of pAMPK Thr172 (62 kDa), AMPK (62 kDa), and β-actin (42 kDa) in HeyA8 cells treated with 100 μM phenformin or 1 mM metformin in normoglycemic or hyperglycemic conditions for 24 h.

Phenformin, another member of the biguanide class of drugs, has been shown to have more potent anti-cancer effects than metformin *in vitro*, likely due to its increased lipophilic nature and greater uptake/transport as compared to metformin [25, 26]. To determine if our findings were biguanide-specific, the effect of phenformin on ovarian cancer growth and AMPK activation was evaluated in normo- and hyperglycemic cell culture conditions. Like metformin, the ability of phenformin to inhibit cell viability was significantly reduced in hyperglycemic (25 mM) as opposed to normoglycemic (5.5 mM) conditions (Figure 3A). In addition, increasing glucose levels dose-dependently inhibited phenformin response (Figure 3B). Treatment with phenformin at one-tenth of the concentration of metformin (100 μM phenformin *vs*. 1 mM metformin) led to a robust increase in pAMPK in 5.5 mM glucose, but only a slight effect in 25 mM glucose (Figure 3C), suggesting that, similar to metformin, lower doses of phenformin induce anti-ovarian cancer effects in normoglycemic conditions.

### Increased glycolytic flux decreases metformin sensitivity in hyperglycemic conditions

**Figure 4:**
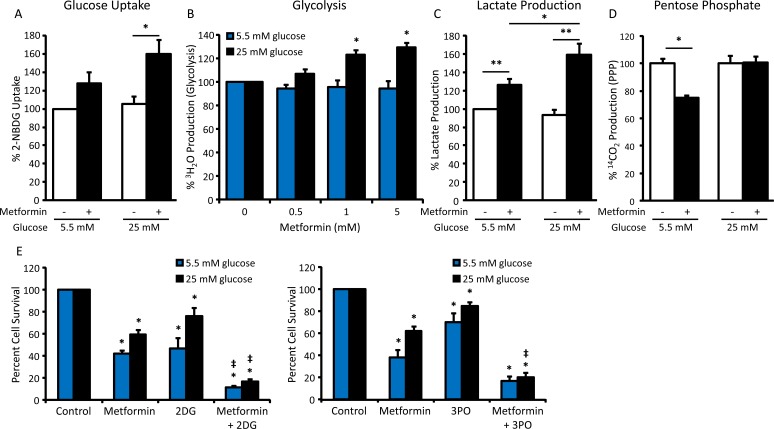
Increased glycolytic flux impairs metformin sensitivity in hyperglycemic conditions **A.** Glucose uptake assay measuring 2-NBDG fluorescence in HeyA8 cells treated with 5 mM metformin in normoglycemic (5.5 mM glucose) or hyperglycemic (25 mM glucose) conditions for 24 h, normalized to protein concentration. Values are mean ± SEM, *n* = 4. *p < 0.05 between the indicated values. **B.** Glycolysis assay measuring the release of ^3^H_2_O from HeyA8 cells treated with 0-5 mM metformin in normoglycemic or hyperglycemic conditions for 24 h, normalized to protein concentration. Values are mean ± SEM, *n* = 4. *p < 0.001 *vs*. hyperglycemic control. **C.** Lactate assay in HeyA8 cells treated with 5 mM metformin in normoglycemic or hyperglycemic conditions for 24 h, normalized to protein concentration. Values are mean ± SEM, *n* = 4. *p < 0.05 or **p < 0.01 between the indicated values. **D.** Pentose phosphate pathway assay measuring the release of ^14^CO_2_ from HeyA8 cells treated with 5 mM metformin in normoglycemic or hyperglycemic conditions for 24 h. Values are mean ± SEM, *n* = 3 in a representative experiment. *p < 0.005 between the indicated values. **E.** Viability assay of HeyA8 cells treated with 5 mM metformin +/− glycolytic inhibitors 2DG (1 mM) or 3PO (10 μM) in normoglycemic or hyperglycemic conditions for 72 h. Values are mean ± SEM, *n* ≥ 3. *p < 0.05 *vs*. normoglycemic or hyperglycemic control, ‡*p* < 0.05 *vs*. treatment with metformin alone.

To explore the mechanism by which cells in hyperglycemic conditions escape the cytotoxic and AMPK-activating effects of biguanides, glucose metabolism was evaluated. A significant increase in glucose uptake was noted following metformin treatment in 25 mM glucose but not in 5.5 mM glucose (Figure 4A). In addition, metformin treatment resulted in a dose-dependent increase in glycolytic flux in hyperglycemic conditions, which was not observed in normoglycemic conditions (Figure 4B). Consistent with the effects on glucose uptake and glycolysis, increased production of lactate as a glycolytic output was observed following metformin treatment, with the greatest increase in lactate production occurring in hyperglycemic conditions (Figure 4C). Interestingly, pentose phosphate pathway (PPP) activity was suppressed by metformin treatment in normoglycemic but not hyperglycemic conditions, suggesting the necessity of glucose utilization for energy production rather than anabolic processes in normoglycemic conditions (Figure 4D).

Next, we asked whether inhibiting glycolysis would improve metformin sensitivity in hyperglycemic conditions. To test this, cells were treated with metformin plus the glycolytic inhibitors 2-deoxy-D-glucose (2DG) or 3-(3-pyridinyl)-1-(4-pyridinyl)-2-propen-1-one (3PO), which inhibit the activities of hexokinase and 6-phosphofructo-2-kinase/fructose-2,6-bisphosphatase-3 (PFKFB3), respectively [27, 28]. In the presence of either glycolytic inhibitor, cells in hyperglycemic conditions were sensitized to metformin; the combined treatment also increased the toxicity of metformin in normoglycemic conditions (Figure 4E). Together, these findings suggest that in hyperglycemic conditions ovarian cancer cells escape the effects of metformin through activation of glycolysis.

### Differential effects of metformin in normo- and hyperglycemic conditions are mediated by c-Myc

**Figure 5:**
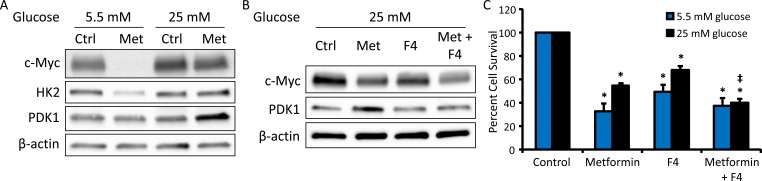
c-Myc inhibition restores metformin sensitivity in hyperglycemic conditions **A.** Western blot of c-Myc (57-65 kDa), HK2 (102 kDa), PDK1 (47 kDa), and β-actin (42 kDa) in HeyA8 cells treated with 0 or 5 mM metformin in normoglycemic (5.5 mM glucose) or hyperglycemic (25 mM glucose) conditions for 48 h. **B.** Western blot of c-Myc (57-62 kDa), PDK1 (47 kDa), and β-actin (42 kDa) in HeyA8 cells treated with 5 mM metformin +/− c-Myc inhibitor 10058-F4 (F4, 100 μM) in hyperglycemic conditions. **C.** Viability assay of HeyA8 cells treated with 5 mM metformin +/− the c-Myc inhibitor 10058-F4 (F4, 100 μΜ) in normoglycemic or hyperglycemic conditions for 72 h. Values are mean ± SEM, *n* ≥ 3. *p < 0.001 *vs*. normoglycemic or hyperglycemic control, ‡*p* < 0.05 *vs*. treatment with metformin alone.

c-Myc is an important oncogenic transcription factor which regulates the expression of many enzymes and nutrient transporters involved in cellular metabolic processes [29]. Since modulation of c-Myc expression can alter glycolytic output to enable metabolic adaptation [30], we examined the effect of metformin treatment on c-Myc protein levels in differential glycemic conditions. c-Myc expression was strongly inhibited by metformin treatment in normoglycemic conditions, while this effect was largely attenuated in hyperglycemic conditions (Figure 5A). Reflecting the decrease in c-Myc expression, expression of the c-Myc transcriptional target hexokinase-2 (HK2), the enzyme which phosphorylates glucose to glucose-6-phosphate, was also suppressed following metformin treatment in normoglycemic conditions (Figure 5A).

Pyruvate dehydrogenase kinase (PDK1) is a c-Myc transcriptional target which acts to inhibit the activity of pyruvate dehydrogenase (PDH), shifting glycolytic output towards production of lactate rather than acetyl-CoA, resulting in increased glycolytic flux and decreased mitochondrial metabolism [30]. Interestingly, while largely unaffected by metformin in normoglycemic conditions, a robust increase in PDK1 expression was observed following metformin treatment in hyperglycemic conditions (Figure 5A). Expression of other putative c-Myc targets, including the glucose transporter GLUT1 and glycolytic enzymes pyruvate kinase (PKM2) and lactate dehydrogenase (LDH), was not altered by metformin treatment in either glycemic condition (Supplemental Figure S1).

To further understand the role of c-Myc in metformin response, cells were treated with a c-Myc inhibitor, 10058-F4 (denoted as F4) [31], alone or in combination with metformin. In hyperglycemic conditions, the addition of F4 to metformin inhibited the induction of PDK1 expression (Figure 5B). In a cell viability assay, addition of F4 also increased the cytotoxic effect of metformin in hyperglycemic conditions. In fact, co-treatment with F4 eliminated the difference in metformin response between hyperglycemic and normoglycemic conditions. In contrast, addition of the c-Myc inhibitor did not enhance metformin response in normoglycemic conditions (Figure 5C), consistent with the strong reduction in c-Myc expression observed following metformin treatment in these conditions. Overall, these results suggest that, in hyperglycemic conditions, the inability of metformin to inhibit c-Myc expression allows for increased PDK1 expression and aerobic glycolysis, which facilitates metformin resistance.

### Metformin response is inhibited in cell lines with *MYC* gene amplification

**Figure 6:**
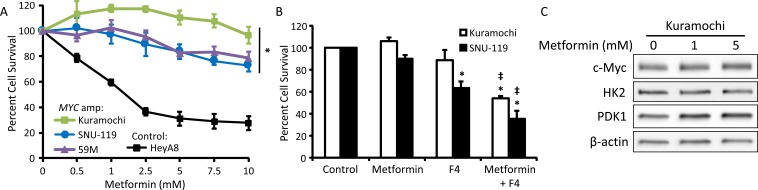
*MYC* gene amplification reduces metformin sensitivity **A.** Three cell lines with *MYC* amplification (Kuramochi, SNU-119, and 59M) and control cells without *MYC* amplification (HeyA8) were treated with 0-10 mM metformin in normoglycemic (5.5 mM glucose) conditions for 72 h and viability was measured using an MTT assay. Values are mean ± SEM, *n* = 3. *p < 0.05 *vs*. HeyA8. **B.** Viability assay of Kuramochi and SNU-119 cells treated with 5 mM metformin +/− the c-Myc inhibitor 10058-F4 (F4, 100 μΜ) in normoglycemic conditions for 72 h. Values are mean ± SEM, *n* ≥ 3. *p < 0.01 *vs*. control, ‡*p* < 0.001 *vs*. treatment with metformin alone. **C.** Western blot of c-Myc (57-65 kDa), HK2 (102 kDa), PDK1 (47 kDa), and β-actin (42 kDa) in Kuramochi cells treated with 0-5 mM metformin in normoglycemic conditions for 48 h.

To further evaluate the impact of c-Myc on metformin sensitivity, metformin response in normoglycemic conditions (5.5 mM glucose) was assessed in three ovarian cancer cell lines with *MYC* gene amplification (Kuramochi, SNU-119, and 59M) and one cell line without *MYC* amplification (HeyA8) [32]. As compared to the cell line without *MYC* amplification (HeyA8), the cytotoxicity of metformin was lower in the three cell lines with *MYC* amplification (Figure 6A). Treatment of *MYC*-amplified cells (Kuramochi and SNU-119) with the combination of metformin and a c-Myc inhibitor (10058-F4, denoted as F4) led to increased metformin sensitivity (Figure 6B). Furthermore, treatment of a *MYC*-amplified cell line (Kuramochi) with metformin in normoglycemic conditions had no effect on c-Myc or HK2 expression, while PDK1 expression was increased (Figure 6C), in direct contrast to our findings in a cell line without *MYC* amplification (HeyA8, Figure 5A). Together, these results suggest that maintenance of c-Myc expression, whether through hyperglycemia or gene amplification, inhibits metformin response.

### Hyperglycemia inhibits response to metformin in an ovarian cancer mouse model

**Figure 7:**
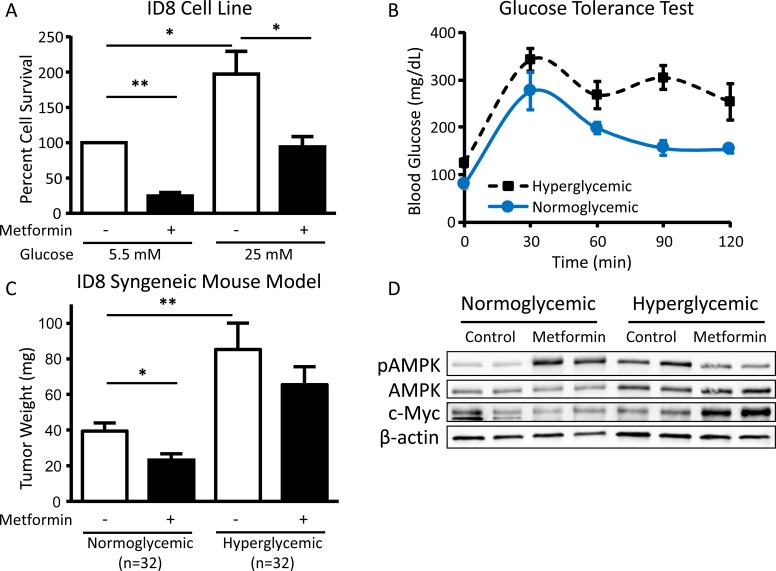
Hyperglycemia inhibits the cytotoxic effect of metformin *in vivo* **A.** Viability of ID8 cells treated *in vitro* with 5 mM metformin in normoglycemic (5.5 mM glucose) or hyperglycemic (25 mM glucose) conditions for 72 h. Values are mean ± SEM, *n* = 3. *p < 0.05 or **p < 0.0001 between the indicated values. **B.** Glucose tolerance test (0-120 min) performed on normoglycemic or hyperglycemic C57BL/6J mice following intraperitoneal injection of 2 g/kg D-glucose. Blood glucose levels were significantly different between normoglycemic and hyperglycemic mice at baseline (0 min) and 90 min post-injection of D-glucose (*p* < 0.01). **C.** Mean tumor weight in normoglycemic or hyperglycemic C57BL/6J mice injected orthotopically with ID8 ovarian cancer cells in the ovarian bursa and treated with intraperitoneal metformin (200 mg/kg) or placebo (PBS) daily. *p < 0.05 or **p < 0.01 between the indicated values. **D.** Western blot of pAMPK Thr172 (62 kDa), AMPK (62 kDa), c-Myc (57-65 kDa), and β-actin (42 kDa) in tumor lysates from normoglycemic or hyperglycemic mice treated with placebo or metformin (*n* = 2 from each group).

In preparation for an *in vivo* experiment, the impact of metformin on the viability of the ID8 mouse ovarian cancer cell line was evaluated *in vitro*. Like the human ovarian cancer cell lines, ID8 cells exhibited decreased sensitivity to metformin treatment in hyperglycemic conditions as compared to normoglycemic conditions (Figure 7A). To evaluate the effect of glycemic conditions on metformin efficacy *in vivo*, hyperglycemia and glucose intolerance were induced in a syngeneic mouse model of ovarian cancer (Figure 7B). Using a cancer prevention strategy (Supplemental Figure S2), the effect of metformin on tumor burden was compared to placebo. At the time of sacrifice, hyperglycemic mice had significantly increased tumor burden as compared to normoglycemic mice and metformin treatment did not significantly reduce tumor weight. In contrast, normoglycemic mice treated with metformin had significantly reduced tumor weight compared to placebo controls (Figure 7C). Complementing the *in vitro* findings, analysis of tumors showed that metformin-treated hyperglycemic mice had decreased AMPK phosphorylation as compared to placebo-treated mice. In contrast, tumors from metformin-treated normoglycemic mice had increased AMPK phosphorylation as compared to placebo-treated controls. Interestingly, c-Myc expression was increased in metformin-treated hyperglycemic mice, suggesting that compensatory pathways similar to those found *in vitro* also occur *in vivo* (Figure 7D).

## DISCUSSION

**Figure 8:**
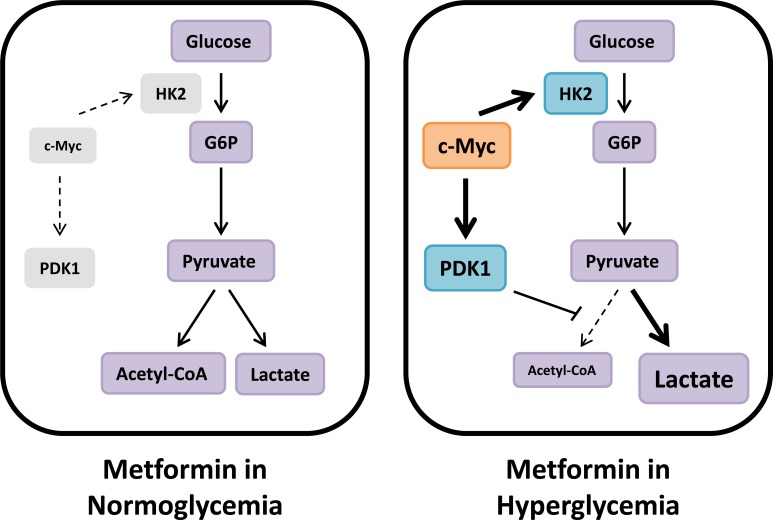
Metabolic compensation in hyperglycemia reduces metformin sensitivity We hypothesize that cancer cells in hyperglycemic conditions are less sensitive to the energetic stress induced by metformin due to compensatory upregulation of glycolysis mediated by c-Myc. Under these conditions, metformin treatment results in increased PDK1 expression, thereby inhibiting PDH activity and shuttling glycolytic output towards lactate production. In normoglycemic conditions, however, c-Myc expression is inhibited by metformin treatment, and this survival-promoting metabolic mechanism does not occur.

Although metformin has shown significant promise as an anti-cancer therapeutic in preclinical studies, concerns remain about the translation of these findings - especially those utilizing high doses of metformin - to potential clinical efficacy in patients without diabetes. In this study, we demonstrate that low doses of metformin inhibit ovarian cancer cell viability and activate AMPK when tested under physiologic normoglycemic conditions (5.5 mM glucose). Furthermore, we show that, in the setting of hyperglycemia, cancer cells undergo a compensatory increase in glycolysis that is likely mediated by c-Myc activity (Figure 8). In support of the *in vitro* findings, in a syngeneic mouse model, metformin treatment resulted in a greater reduction in tumor weight in normoglycemic mice as compared to hyperglycemic mice, with suppression of AMPK phosphorylation and induction of c-Myc expression observed in the hyperglycemic mice treated with metformin.

Only by evaluating metformin in glucose conditions that more closely reflect normal physiology will we begin to get a comprehensive understanding of the molecular mechanisms mediating the drug's anti-cancer effects. We demonstrate that ovarian cancer cells specifically in hyperglycemic conditions, but not normoglycemic conditions, are able to escape the cytotoxic effects of metformin by increasing glucose uptake and glycolytic flux, and that addition of a glycolytic inhibitor (2DG or 3PO) improves metformin response. These results are consistent with findings in other cancers indicating that metformin increases extracellular acidification [33] and that 2DG enhances the cytotoxic effects of metformin [34, 35]. Furthermore, under normoglycemic conditions, we were able to identify a suppressive effect of metformin on the pentose phosphate pathway; a finding that, to our knowledge, has not yet been reported and likely contributes to the anti-cancer effects of metformin.

Mechanistically, we demonstrate that sustained c-Myc expression facilitates metformin resistance. Consistent with reports in breast and prostate cancer indicating that metformin suppresses c-Myc expression through miRNA-mediated inhibition [36] or post-translational modification [37], we show that metformin reduces c-Myc expression in ovarian cancer specifically under normoglycemic conditions. In contrast, in hyperglycemic conditions, metformin does not reduce c-Myc expression, instead resulting in increased PDK1 expression and glycolytic flux. Consistent with our findings, addition of a PDK1 inhibitor, dichloroacetate, enhanced metformin response in prostate cancer cells [38]. Further supporting a role for c-Myc in metformin resistance, ovarian cancer cell lines with *MYC* gene amplification were relatively insensitive to metformin treatment even in normoglycemic conditions. Together, these findings not only explain the attenuated effect of metformin in hyperglycemic conditions, but also suggest that, clinically, *MYC* amplification in patient tumors might serve as a predictive biomarker of metformin response.

As outlined above, the molecular mechanisms mediating the anti-cancer effects of metformin are multi-faceted and context-dependent. However, clinically, the most important question is: Will metformin, at physiologically attainable doses, have anti-cancer effects in patients without diabetes? In this report we show that, compared to prior studies [17, 18], 10-20-fold lower doses of metformin are effective if cell culture conditions are normoglycemic. As the epidemiological evidence suggesting potential anti-cancer effects of metformin is from patients using metformin as treatment for diabetes, some argue that metformin will not work as a cancer therapeutic in patients without diabetes. Our findings suggest the opposite. Using a hyperglycemic syngeneic mouse model we showed that metformin's inhibition of ovarian cancer growth was greatest in normoglycemic mice and, in fact, metformin did not significantly reduce tumor growth under conditions mimicking hyperglycemia in poorly controlled diabetes. Complementing our *in vitro* findings, c-Myc expression was strongly induced in hyperglycemic mice treated with metformin, reflective of compensatory metabolic changes promoting cancer cell survival.

In summary, we report that low doses of metformin can inhibit ovarian cancer cell growth if cell culture conditions are normoglycemic, and that metformin sensitivity is reduced in hyperglycemic conditions. Mechanistically, we demonstrate that c-Myc-mediated compensatory metabolic changes inhibit response to metformin and that the anti-cancer effects of metformin can be restored through the addition of either a glycolytic or c-Myc inhibitor. Our findings, both *in vitro* and *in vivo,* using a hyperglycemic mouse model, support the hypothesis that metformin will have anti-cancer benefits in non-diabetic patients. However, this question can ultimately only be answered through the prospective clinical testing of metformin as adjuvant treatment in cancer patients without diabetes. Preclinical reports in ovarian cancer indicate that metformin increases response to carboplatin and paclitaxel chemotherapy [17, 39-42]. Building on these findings, several clinical trials are underway for gynecologic malignancies [6], including our ongoing trial of standard chemotherapy with or without metformin as up-front treatment for ovarian cancer (NCT02122185,https://clinicaltrials.gov/). Equally important to prospective testing, future evaluation of the molecular mechanisms of action mediating metformin's anti-cancer effects should only be undertaken with serious consideration of the impact of glucose levels, perhaps aided by the utilization of innovative cell culture techniques to increase the reliability of *in vitro* experiments [43]. Ultimately, understanding the effects of the nutrient environment and cellular metabolic regulation on metformin response will aid the design and interpretation of preclinical experiments and inform the clinical use of metformin in the treatment of ovarian and other cancers.

## MATERIALS AND METHODS

### Reagents and cell lines

The HeyA8, Tyk-nu, DOV13, and ID8 ovarian cancer cell lines were provided by Dr. Gordon Mills, Dr. Kenjiro Sawada, Dr. Maria Barbolina, and Dr. Katherine Roby, respectively. The Kuramochi, SNU-119, and 59M ovarian cancer cell lines were purchased from the Japanese Collection of Research Bioresources Cell Bank, the Korean Cell Line Bank, and the European Collection of Cell Cultures, respectively. All cell lines were authenticated by IDEXX BioResearch (Columbia, MO). Metformin (1,1-dimethylbiguanide hydrochloride), phenformin (phenethylbiguanide hydrochloride), and 2DG were obtained from Sigma-Aldrich (St. Louis, MO). 3PO and 5-[(4-ethylphenyl)methylene]-2-thioxo-4-thiazolidinone (10058-F4) were purchased from Calbiochem/Millipore (Billerica, MA) and Cayman Chemical Company (Ann Arbor, MI), respectively. The pAMPK Thr172 (40H9), AMPK (23A3), GAPDH (14C10), pS6 Ser240/244, S6 (54D2), pACC Ser 79, c-Myc (D84C12), PDK1 (C47H1), HK2 (C64G5), and HRP-linked goat anti-rabbit and horse anti-mouse antibodies were purchased from Cell Signaling Technology (Beverly, MA). The ACC and β-actin (AC-15) antibodies were from Millipore and Sigma-Aldrich, respectively.

### Isolation of primary ovarian cancer cells from ascites

Ascites were collected from a chemotherapy-naïve ovarian cancer patient via paracentesis under a protocol approved by the University of Chicago Institutional Review Board. Ascites (100 mL) were immediately centrifuged for 5 min at 500 × g. The resulting cell pellet was re-suspended in PBS and passed through a 40 μm nylon mesh cell strainer to enrich for cancer cell spheroids. Cells were re-suspended in DMEM containing 10% FBS, non-essential amino acids, and vitamins and incubated in tissue culture plasticware for 6 h to remove remaining immune and mesothelial cells via differential adhesion. The supernatant containing cancer cells was transferred to new tissue cultureware and cells were allowed to adhere for 72 h prior to use in experiments.

### Cell viability assays

Cells were plated in 96-well plates in quintuplicate overnight and subsequently treated as indicated. Cell viability was determined via MTT assay with 0.5 mg/mL thiazolyl blue tetrazolium bromide (Sigma-Aldrich), as previously described [44]. For ID8 cells, cell number was determined by cell counting and trypan blue exclusion.

### Immunoblotting

Cells were plated in 60 mm dishes overnight in DMEM containing 5.5 or 25 mM glucose and subsequently treated as indicated. Lysates were prepared and immunoblotting was performed as previously described [17].

### Long-term culture in normoglycemic or hyperglycemic conditions

HeyA8 cells were cultured in DMEM containing 5.5 or 25 mM glucose. Cells were passaged every three days with media changed daily to maintain glucose levels. Glucose concentrations were measured daily using a FreeStyle glucometer (Abbott Laboratories, Abbott Park, IL). Following >2 weeks of culture in the indicated levels of glucose, cells were subjected to cell viability and western blot assays as described.

### Glucose uptake assay

Cells were plated in black 96-well plates overnight in DMEM containing 5.5 or 25 mM glucose and subsequently treated with 5 mM metformin for 24 h. Glucose uptake was determined using a fluorescently labeled glucose analog, 2-deoxy-2-[(7-nitro-2,1,3-benzoxadiazol-4-yl)amino]-D-glucose (2-NBDG; Cayman Chemical Company) [45], according to manufacturer's instructions. Relative fluorescence was normalized to protein concentration.

### Glycolytic flux assay

Glycolytic flux was assayed by quantifying the ^3^H_2_O produced from [5-^3^H]glucose through the enolase step of glycolysis, as described in [46, 47] with minor modifications. Cells were plated in 12-well plates overnight in DMEM containing 5.5 or 25 mM glucose and subsequently treated with 0.5, 1, or 5 mM metformin for 24 h. Following treatment with metformin, all cells were changed to fresh media containing 1 μCi/mL [5-^3^H]glucose (PerkinElmer, Waltham, MA) for 1 h. The media was then collected and centrifuged for 5 min at 8,000 rpm. To separate the ^3^H_2_O produced by the cells, 150 μL of media were placed in a tube surrounded by 1 mL H_2_O in a closed system and allowed to equilibrate for 48 h at 37°C. The tube containing media was then removed and ^3^H was quantified using a Tri-Carb scintillation counter (Packard/PerkinElmer), as a measure of glycolytic flux. Counts were normalized to protein concentration.

### Pentose phosphate pathway assay

Pentose phosphate pathway flux was assayed by quantifying the ^14^CO_2_ produced from [1-^14^C]glucose and released with the concomitant generation of ribulose-5-phosphate, as described in [48] with minor modifications. Cells were plated in T-25 flasks in DMEM containing 5.5 or 25 mM glucose and allowed to attach overnight. To measure the ^14^CO_2_ released, a well (Kimble Chase, Vineland, NJ) containing filter paper saturated in 10 M KOH was inserted into each flask and the cells were treated with 5 mM metformin in DMEM containing 5.5 or 25 mM glucose. The cells were simultaneously labelled with 3 μCi/mL [1-^14^C]glucose. After 24 h, 1 mL 3 N acetic acid was added to each flask and incubated at room temperature for 1 h for complete release of ^14^CO_2_ from the media. The filter paper was then removed from the chamber and ^14^C was quantified using a Tri-Carb scintillation counter (Packard/PerkinElmer), as a measure of pentose phosphate pathway flux.

### Lactate assay

Production of lactate was determined using the EnzyChrom™ L-Lactate Assay Kit (BioAssay Systems, Hayward, CA). Cells were plated in 96-well plates overnight in DMEM containing 5.5 or 25 mM glucose and subsequently treated with 5 mM metformin for 24 h. Following treatment with metformin, medium was collected from the cells and lactate levels were assayed via colorimetric detection at 595 nm according to manufacturer's instructions. Absorbance was normalized to protein concentration.

### Hyperglycemic syngeneic ovarian cancer mouse model

A model of hyperglycemia was generated by feeding 5 week old female C57BL/6J mice (Jackson Laboratory, Bar Harbor, ME) a 60% kCal fat diet (Harlan Teklad, Indianapolis, IN) for 4 months. Normo- and hyperglycemic mice were then treated with metformin (200 mg/kg/day) or placebo (PBS) intraperitoneally for 3 weeks. ID8 mouse ovarian cancer cells (1.2×10^6^) were injected orthotopically into the ovarian bursa and treatment with metformin or placebo was continued for 12 weeks before the mice were sacrificed. Glucose tolerance tests were performed following a 16 h fast by injecting 2 g/kg D-glucose (Sigma-Aldrich) intraperitoneally and measuring blood glucose levels with a FreeStyle glucometer (Abbott Laboratories). Mean tumor weight in the ovary was compared between normo- and hyperglycemic mice treated with metformin or placebo. Tumors were homogenized in RIPA buffer and protein expression was analyzed by western blot. All animal procedures were approved by the Institutional Animal Care and Use Committee of the University of Chicago.

## SUPPLEMENTARY MATERIAL FIGURES


