# Supplementary material for: Hyperglycemia-induced metabolic compensation inhibits metformin sensitivity in ovarian cancer
Source: Oncotarget. 2015 Jun 19;6(27):23548–60. doi: 10.18632/oncotarget.4556 (PMC4695136; doi:10.18632/oncotarget.4556)
Supplement: Supplementary file 1 [file oncotarget-06-23548-s001.pdf]

Hyperglycemia-induced metabolic compensation inhibits metformin sensitivity in ovarian cancer

Supplementary Material

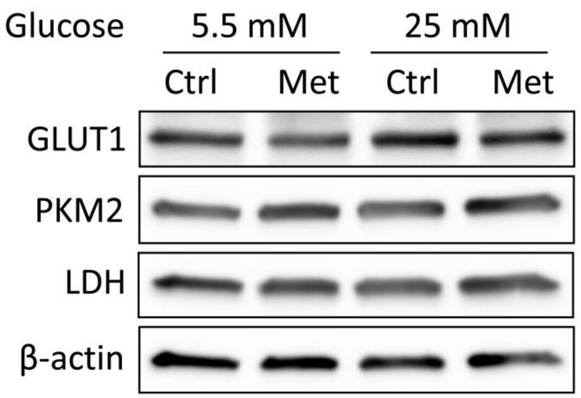

Supplemental Figure S1: Putative c-Myc targets not affected by metformin treatment.
Western blot of GLUT1 (Millipore; 54 kDa), PKM2 (Cell Signaling Technology, D78A4; 60 kDa), LDH (Cell Signaling Technology, C4B5; 37 kDa), and β-actin (42 kDa) in HeyA8 cells treated with 0 or 5 mM metformin in normoglycemic (5.5 mM glucose) or hyperglycemic (25 mM glucose) conditions for 48 h.

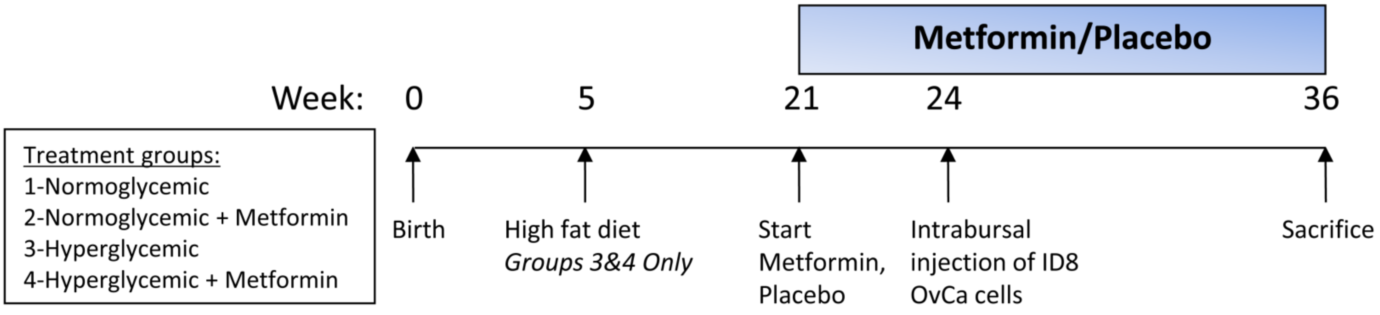

Supplemental Figure S2: Mouse model design.
Five week old female C57BL/6J mice were fed a 60% kCal fat or normal chow diet for 4 months. Mice were then treated with placebo (PBS) or metformin (200 mg/kg/day) injected intraperitoneally for 3 weeks in a cancer prevention treatment stragey. ID8 mouse ovarian cancer cells were orthotopically injected into the ovarian bursa and treatment (metformin or placebo) was continued for 12 weeks before the mice were sacrificed.
